# *SASfit*: a tool for small-angle scattering data analysis using a library of analytical expressions

**DOI:** 10.1107/S1600576715016544

**Published:** 2015-09-20

**Authors:** Ingo Breßler, Joachim Kohlbrecher, Andreas F. Thünemann

**Affiliations:** a BAM Federal Institute for Materials Research and Testing, Unter den Eichen 87, Berlin, 12205, Germany; b Laboratory for Neutron Scattering, PSI Paul Scherrer Institute, Villigen, CH-5232, Switzerland

**Keywords:** small-angle X-ray scattering, small-angle neutron scattering, curve fitting, nanotechnology, nanoparticles, polymers

## Abstract

*SASfit* is one of the mature programs for small-angle scattering data analysis and has been available for many years. This article describes the basic data processing and analysis workflow along with recent developments in the *SASfit* program package (version 0.94.6). They include (i) advanced algorithms for reduction of oversampled data sets, (ii) improved confidence assessment in the optimized model parameters and (iii) a flexible plug-in system for custom user-provided models. A scattering function of a mass fractal model of branched polymers in solution is provided as an example for implementing a plug-in. The new *SASfit* release is available for major platforms such as Windows, Linux and MacOS. To facilitate usage, it includes comprehensive indexed documentation as well as a web-based wiki for peer collaboration and online videos demonstrating basic usage. The use of *SASfit* is illustrated by interpretation of the small-angle X-ray scattering curves of monomodal gold nanoparticles (NIST reference material 8011) and bimodal silica nanoparticles (EU reference material ERM-FD-102).

## Abstract 2

A computer program to perform small-angle X-ray and neutron scattering data evaluation is presented.

## 1. Introduction   

With an increasing number of applications for small-angle scattering (SAS) experiments, for example in materials science, structural biology and analysis of soft matter, it is important to assist the user to extract structural information from measurements. There are a variety of approaches to obtain such structural parameters from the data, including model-free analysis, model fitting and inversion methods, several of which have been implemented in available software.

A few well established programs for model-based SAS data analysis are actively maintained. *IRENA*, for example, is suitable for a wide range of sample types (Ilavsky & Jemian, 2009 ▸). *Scatter* is a program geared towards the analysis of two-dimensional data from nano- and mesoscale oriented structures (Förster *et al.*, 2010 ▸). Furthermore, there is the *ATSAS* project, consisting of a comprehensive set of sophisticated tools primarily intended for biological systems (Petoukhov *et al.*, 2012 ▸). Lastly, *McSAS* uses a Monte Carlo algorithm to extract form-free model parameter distributions for disperse sample types (Bressler *et al.*, 2015 ▸). To further increase the availability of generally applicable analysis software, *SASfit* has been developed.

Originally written for users at the Paul Scherrer Institute (PSI), *SASfit* was made available to the general public in 2010. Since then it has been widely adopted and is downloaded more than 2000 times per year (http://sourceforge.net/projects/sasfit/files/stats/timeline?dates=2014-01-01+to+2014-12-31). The program is distributed for the Windows, Linux and MacOS platforms under the conditions of the GPL Open Source License (Version 3).

*SASfit* is a program primarily for model-based analysis of SAS data, with an easy to use interface lowering the barrier of entry. Besides its ease of use, it is very versatile, contains over 200 models for fitting and can be easily extended by the user to include even more. The fitting interface also allows for the construction of compound models which can then be used to fit one or more data sets. Examples of the application of this program include the traceable size determination of gold nanoparticles (Meli *et al.*, 2012 ▸), polymeric nanoparticles (Gleber *et al.*, 2010 ▸) and vesicles (Varga *et al.*, 2014 ▸). More exotic applications include the study of relaxation mechanisms in magnetic colloids by stroboscopic spin-polarized small-angle neutron scattering (SANS) (Wiedenmann *et al.*, 2011 ▸), simultaneous fitting of up to 70 measurements from contrast variation experiments (Kohlbrecher *et al.*, 2006 ▸; Vavrin *et al.*, 2009 ▸) and analysing the (model-free) integral structural parameters of 400 scattering curves for creating local contrast in SANS by dynamic nuclear polarization (van den Brandt *et al.*, 2007 ▸).

This is the first paper describing *SASfit*. It details data import and data set reduction, model configuration, and curve fitting. Advice is given on the interpretation of fit parameter confidence bounds, and basic functions for data export and batch processing are explained. Two examples show the analysis of the small-angle X-ray scattering (SAXS) curves of monomodal gold nanoparticles and bimodal silica nanoparticles. Finally, we present the program’s capability of incorporating user-defined model functions.

## 2. Data import   

### 2.1. Data read-in   

*SASfit* loads three-column ASCII data files containing the magnitude of the scattering vector *q*, intensity 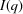 and uncertainty on the intensity 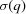. The column content (*q*, *I* or 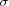) and the ordering of columns can be indicated in the data import dialogue. Note that *SASfit* is unit agnostic by design, with all calculations performed in the units as supplied. Within a file, anything that can be interpreted as floating point numbers is treated as data; header lines should, therefore, be specifically omitted. *SASfit* requires uncertainties for the fitting process, but if none can be provided *SASfit* estimates them from the smoothness of the curve.

A single data set can be loaded for basic analysis using the ‘Single Data File’ menu. After a single data file has been loaded, more data can be added to the same data set using the subsequent ‘Merge Files’ menu. In this way, measurements from the same sample spanning different *q* ranges can be merged to form a single data set. Furthermore, data sets of the same sample measured under different conditions (*e.g.* contrast variation or concentration variation) can be loaded using the ‘Multiple data set’ menu. This allows for the simultaneous fitting of multiple data sets, thus making additional dimensions of information available in the same fit.

Before data fitting, it is wise to investigate the data plot and its uncertainty estimates (‘errors’). At high *q*, the intensity tends to be low with a high degree of uncertainty. Accordingly, high-*q* data of radially averaged one-dimensional data tend to contribute little to the overall goodness-of-fit, defined by the reduced chi-squared (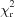) value (Bevington & Robinson, 2003 ▸):
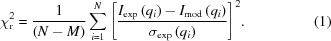



When observing high uncertainties on a large number of data points, a data averaging step should be performed on these data points to prevent instabilities and large calculation times. For quick evaluation, however, it is an option to initially ignore a section of points in the high-*q* region. Furthermore, it might be necessary to skip invalid data points, which may originate from, for example, improper masking of the beam stop, edge effects in radial integration or parasitic scattering contributions.

For these reasons, additional options are provided during data import, which allow the user to specify the *q* range for each data file individually when merging data files. For final data analysis it is recommended, however, to bin the data properly to reduce the number of data points and to improve the data and the data statistics. The adaptive averaging method presented in the next section can be used for this purpose.

### 2.2. Data reduction   

An important step in preparing data for analysis is to reduce the number of data points of oversampled data sets. A reduced number of data points will increase fitting speed proportionally and improve fitting stability. These data reduction options are available in the ‘Merge Files’ overview of *SASfit* and can be applied for each data file individually. Of the three available methods, only the third is recommended for use for data fitting. The first two, which merely omit data points, are intended for data evaluation purposes only.

**Figure 1:**
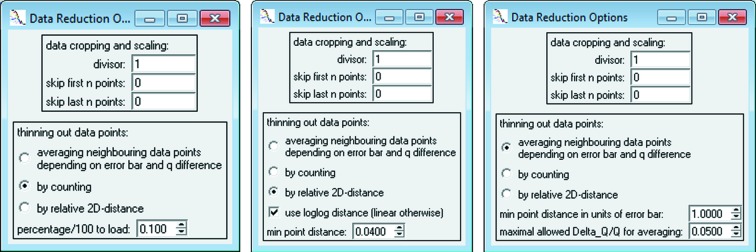
Data reduction window, providing three different methods for reducing the number of data points. Method one (leftmost figure) skips data points by a count ratio [see equation (2)]. For fast data fitting of 10^3^ data points, a typical fraction of 0.1 is recommended. Method two (middle figure) is a fast method for data with distinctive curve features [see equation (3)]. Method three (right-hand figure) averages data points adaptively according to intensity and *q* spread [see equation (6)].

Method one is a simple and straightforward thinning out of data points according to
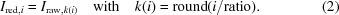
The user specifies a ratio of the original data points to keep. For example, 90% of the points can be omitted in the fit by specifying 0.1, as shown in the left-hand panel of Fig. 1 ▸. This rough method is suitable when working with high-density SAXS data of several thousand data points and when very fast fitting results are desired to get an initial overview, for example during an experiment.

The second method preserves scattering curve characteristics better than the first. It maintains a user-defined distance 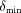 between data points by utilizing Pythagoras’ theorem in linear or logarithmic two-dimensional space and skips those points which are less far away in accordance with
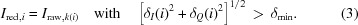
In linear context, 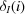 and 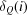 are calculated by
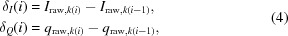
and in logarithmic context by
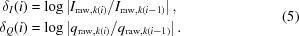



The third, and most recommended, method for data reduction averages neighbouring data points locally, on the basis of the difference in intensity and width in *q* space (see right-hand panel of Fig. 1 ▸). Each local interval 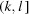 is determined adaptively so that it contains all points *n* which fulfil the following condition:
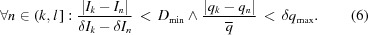
The parameter 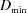 restricts the intensity difference within an interval proportional to the associated uncertainties. Additionally, the maximum width of an interval relative to its position in *q* space is scaled by the second parameter, 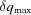. This results in a narrow spacing between data points at low *q* and a wide spacing at high *q*. Both conditions have to be fulfilled by neighbouring data points to fall within an interval and thus to allow calculation of an average. This last method retains information on sharp features, while averaging data points.

It is important to note that the original data are always stored alongside the modified data, and are also stored for traceability in *SASfit* project files along with the reduced data. Adjusting the data affects the copy of the data used for numerical analysis only. The data selected for analysis can, therefore, be changed at any time.

## 3. Model fitting   

### 3.1. Model configuration   

The main purpose of *SASfit* is to fit a model described by idealized scattering functions to one or more data sets. By minimizing the goodness-of-fit 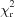 [see equation (1)], through adjustment of the model parameters, the model intensity 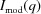 is matched with the measured intensity 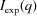. *SASfit* is designed to let the user configure every aspect of 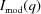, defined as
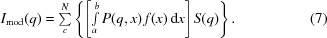



The model intensity is based on the sum of the scattering intensity contributed by different scatterers in the analysed sample. Each scattering contribution *c* consists of a form factor 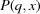 determining the shape of a scatterer. Disperse aspects of shape-similar contributions can be reflected by applying a parameter distribution 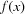, in the range 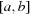, to an arbitrary form factor parameter *x*. Furthermore, *SASfit* allows defining a structure factor 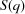 for each contribution, reflecting attraction and repulsion of scatterers in the sample. This model composed of several scattering contributions can be managed in the graphical user interface of the program.

**Figure 2:**
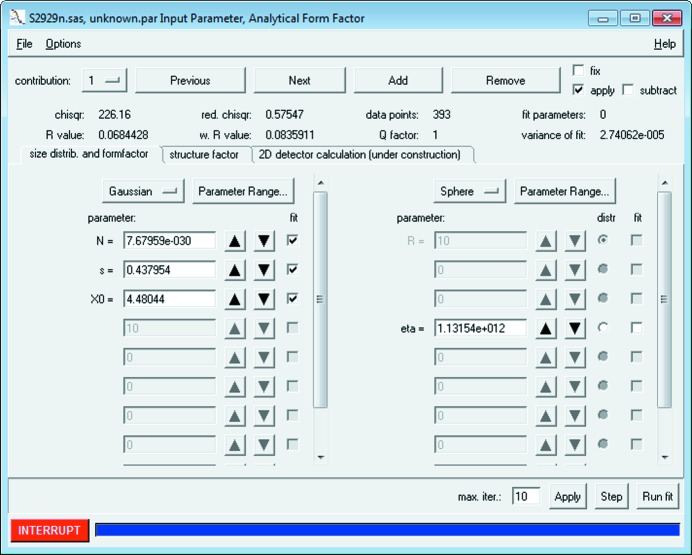
Basic model configuration, consisting of a form factor on the right (in this panel it is a ‘Sphere’ with the scattering contrast of gold nanoparticles ‘eta’ = 1.13 × 10^12^) and a distribution of one selected parameter (‘distr’ column) on the left (here a ‘Gaussian’ distribution of the radius which has a concentration parameter of ‘N’ = 7.6 × 10^−30^, a width parameter ‘s’ = 0.43 and the mean radius parameter ‘X0’ = 4.48 nm. A structure factor can be configured on the second tab. Different scattering contributions can be managed by the top row of buttons.

The model configuration window allows access to the model settings and provides several other options. At the top of the model configuration window in Fig. 2 ▸, there are buttons to ‘Add’ and ‘Remove’ a contribution, as well as switch back and forth to the ‘Next’ and ‘Previous’ scattering contribution. The current contribution is shown in a selection box on the left side of that bar of buttons. Additionally, each contribution can be

(1) disabled but not removed by unchecking the ‘Apply’ checkbox

(2) ‘Fixed’ and thus kept constant during a fit

(3) ‘Subtracted’ from the overall model instead of added by default

When the model and its contributions are configured correctly as desired, the initial model configuration can be plotted against the loaded data by using ‘Apply’.

There are over 200 form factors available in *SASfit*. This list includes the commonly used ‘Sphere’ and associated form factors, but also includes models that do not strictly adhere to the form factor definition, such as the Beaucage unified fit model (Sztucki *et al.*, 2007 ▸) and several models for disordered structures. One typical starting model for fitting data would be the ‘Sphere’ model, coupled with a parameter distribution over the sphere radius *R*. By starting with such a simple model, alternative and typically more complex models can be assessed for their significance against the fit quality of the ‘Sphere’ model. This example is given in Fig. 2 ▸ by using a Gaussian distribution. Depending on the scientific field, either the Gaussian, lognormal or Schulz–Zimm distributions are typical choices for describing the (poly)dispersity of monomodal shape parameter distributions.

Each distribution consists of at least one parameter controlling the position of its maximum and one parameter controlling its width or FWHM, which defines the degree of polydispersity. The monodisperse distribution, being the exception to this, defines only a single parameter value. Nontrivial parameter distributions are integrated by linear subdivision of the integration range, whose extent and granularity are determined adaptively on the basis of the user-provided distribution parameters. Finding a suitable integration range depends on the distribution functions and cannot be handled in an efficient and numerically stable way for all possible functions. Therefore, a fixed set of available parameter distributions is defined in *SASfit*, which cannot (yet) be extended by the user.

Besides form factors and distribution functions, *SASfit* allows consideration of interparticle scattering effects. There are several approximations implemented to allow calculation of such a structure factor: (1) monodisperse approximation [see equation (7)], (2) decoupling approach, (3) local monodisperse approach, (4) partial structure factor and (5) scaling approximation of partial structure factor. It should be noted that some of these structure factor approximations [specifically, numbers (2) and (5)] require knowledge of the scattering amplitude of the form factor, as opposed to ‘just’ the scattering intensity. *SASfit* might return an error if such a structure factor approximation is attempted in combination with a form factor for which the scattering amplitude is not known. Details on the exact formulae are given in the *SASfit* manual (Kohlbrecher & Breßler, 2015 ▸).

Structure factors affect a scattering curve at low *q* values owing to the larger lengths they inherently cover. Here, the residuum of a fit would show oscillations if disregarded particle interactions are significant. In order to assess the influence of a structure factor on the fit of the model configuration and the experimental data, it can be selected and configured in the second tab, ‘structure factor’, of the model configuration window shown in Fig. 2 ▸. In many cases, a simple ‘Hard Sphere’ structure factor would suffice. In its basic configuration, the repulsion radius is set slightly larger than the particle radius but maintains the same order of magnitude, whereas its volume fraction is set to small values such as 0.05 at the start of the fitting procedure.

Before moving on to the least-squares fitting procedure, it is recommended to constrain the fit parameters algorithm to a physically feasible range of parameter values. They can be defined by the ‘Parameter Range’ menu next to each model function for single data set fitting. If no constraints are applied, the fitting procedure may result in no solution or an unrealistic local minimum. Each parameter contains a brief explanation of its meaning, which is shown in a tooltip and at the bottom of the window upon hovering the cursor over the parameter.

### 3.2. Curve fitting workflow   

**Figure 3:**
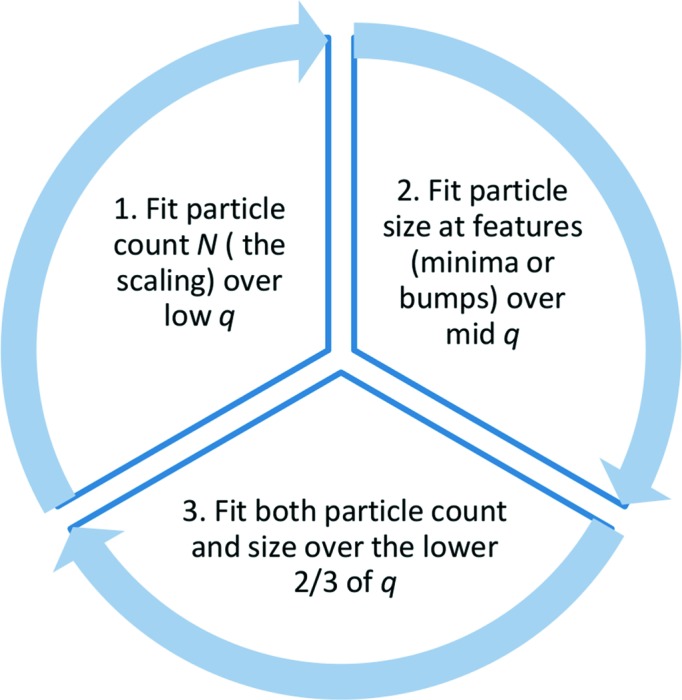
The recommended curve fitting workflow of *SASfit* consists of a three-step cycle.

It is recommended to adjust the fit parameters manually before starting the iterative least-squares optimization, in order for the model intensity to approach or intersect with the data. This helps to prevent instability during the initial iterations. Owing to the minimization exhibiting many local minima it is strongly recommended to optimize the model parameters stepwise. Otherwise, a minimization of all parameters in one step automatically either will not reach the intended best fit or may provide a physically meaningless solution. Therefore, the basic workflow for fitting small-angle scattering data consists of the following steps (as illustrated in Fig. 3 ▸):

(1) The first step of a fit procedure is to match the order of magnitude of the model curve (red) and data intensity (black dots with blue error bars). This can be accomplished by initially fitting the scaling parameter at the beginning of the curve only. The distribution parameter *N* should be used for this purpose, which denotes the number of scattering objects involved in the measurement. Applying ‘Run fit’ finds the optimal value of *N*.

(2) The size of scattering objects is optimized in the second step of curve fitting. The best results are obtained by limiting the fit to the central part of the data where, for example, a first local minimum of the curve can be observed.

(3) Fitting both the scaling parameter and the size parameter at the same time over the first two-thirds of the data further improves the overall quality of the fit.

In a final step, some slight mismatch in the central part of the scattering curve can be optimized by fitting the particle radius together with the distribution width parameter ‘s’ (width of the Gaussian distribution in the example). Steps 1–3 can be repeated, if necessary, until a good overlap of the model curve and the data is obtained.

With increasing value of the size distribution width parameter, the model curve becomes smoother since it represents a broader size range of scattering objects. Because of this smoothing, evidence supporting a particular shape reduces, making it increasingly challenging to distinguish between different shapes coupled with large polydispersities (as many will fit the data to a similar degree). Therefore, care has to be taken in interpretation of broad size distributions which are larger than about 20% of the mean value at σ; external supporting evidence for a particular shape assumption should be provided.

### 3.3. Example 1: disperse gold nanoparticles   

To aid in the discussion of the remaining *SASfit* aspects, such as fit interpretation and reliability, we will first show an application example. By means of this example, necessary considerations for retrieving reliable values for scatterer population from absolute measurements and advice on the interpretation of uncertainties will be provided. In order to demonstrate the reliability of SAS data analysis with *SASfit* we chose a dispersion of gold nanoparticles of the NIST reference material RM-8011 (De Temmerman *et al.*, 2014 ▸; Kaiser & Watters, 2007 ▸).

**Figure 4:**
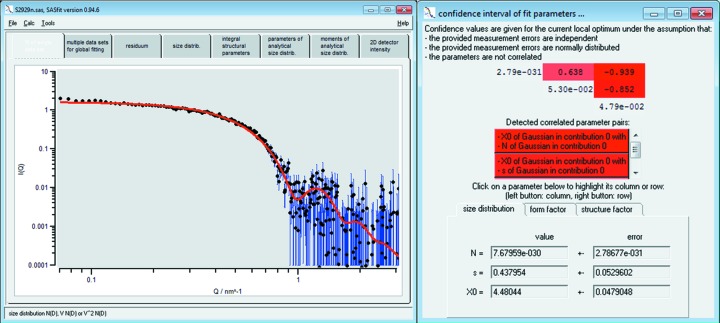
Curve fit of a single data set of gold nanoparticles of the NIST reference material RM-8011 (De Temmerman *et al.*, 2014 ▸; Kaiser & Watters, 2007 ▸) using a model of spheres with Gaussian size distribution and the initial parameters shown in Fig. 2 ▸. The uncertainties of the measured intensity values are displayed as blue lines. The mean radius is *X*_0_ = 4.48 (5) nm and the width of the size distribution is *s* = 0.44 (5) nm. Right-hand figure: covariance matrix and confidence intervals of fit parameters to assess (inter-)correlation of parameters.

A straightforward example in which the presented fit procedure is applied is the determination of the mean radius of spherical particles in solution, the width of their radius distribution and the particle number concentration. We measured RM-8011 for 30 min with a SAXSess instrument from Anton Paar and scaled the data to absolute intensity using water as a primary standard as described by Orthaber *et al.* (2000 ▸). The resulting data have been fitted to a model composed of spheres with a Gaussian size distribution. The result is shown in Fig. 4 ▸ (black dots and red solid curve, respectively, with the uncertainties on the intensity values displayed as vertical blue lines). [The obtained data set is provided as supporting information (GoldS2843.pdh) besides a preconfigured *SASfit* project file (GoldS2843.sas).]

The fit parameters in this example are values for the particle concentration *N*, the mean particle radius *X*_0_ of the assumed Gaussian size distribution and the width of the size distribution *s*. Note that numerous size distributions are provided, including the frequently used Schulz–Zimm (Flory) and lognormal distributions. It is the users’ choice to select the most appropriate one. We recommend commencing with a Gaussian size distribution if no evidence is available to support an alternative size distribution, for example from other methods like electron microscopy or from physical considerations.

The best fit value for the mean radius in our example is *X*_0_ = 4.48 (5) nm and the width of the size distribution is *s* = 0.44 (5) nm. Here, the uncertainties merely denote the standard errors as determined from the least-squares optimization method. These uncertainties can be utilized to determine the combined standard uncertainties from all input quantities (Meli *et al.*, 2012 ▸); however, this can be a tedious procedure which is beyond the scope of this report. As a rule of thumb, the uncertainty of the size parameters from *SASfit* is of the same order of magnitude as the combined standard uncertainties. Another common calculation, possible when the data have been scaled to absolute units, is the determination of the concentration of scatterers in solution.

In order to convert the *N* value to a particle concentration (in number of particles per cm^3^), the units used for absolute intensity, scattering vector and scattering length density should be considered. Here, this conversion factor is 10^42^ as the corresponding units used were cm^−1^, nm^−1^ and cm^−2^:
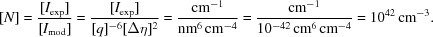
Therefore, in our example, the *N* value of 7.68 (28) × 10^−30^ corresponds to a particle number concentration of 7.68 (28) × 10^12^ cm^−3^ or a molar concentration of 1.28 (5) × 10^−7^ mol l^−1^. To double-check this value, it is recommended to convert the particle number concentration to the mass (and volume) fraction 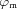 (and 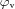, respectively).

For a Gaussian size distribution the mass fraction is 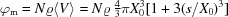, where 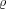 is the density and 〈*V*〉 the mean particle volume. Assuming that the gold particles in our example have the same density as the bulk material (19.30 g cm^−3^), we calculated a mass fraction of 57.37 (209) µg g^−1^. This value is in reasonable agreement with the value of 51.56 (23) µg g^−1^ (2.67 × 10^−4^ vol.%) provided by NIST, as determined by inductively coupled plasma optical emission spectrometry (Kaiser & Watters, 2007 ▸). It should be noted that the uncertainty of the intensity measurements is of the order of 5%, which means that the uncertainty of *N* must be of the same order of magnitude, in other words larger than the least-squares derived uncertainty (Orthaber *et al.*, 2000 ▸). *SASfit* furthermore provides several measures which can be used to assess the quality of the (local) optimization minima found, which will be discussed in the next section.

### 3.4. Fit quality   

The quality of a fit is mainly described by the relevance of the data with respect to the model and its parameters: Would the combination of model and parameters provide the same fit quality for another data set, possibly random data? To answer this question, *SASfit* provides measures that serve as indicators of the fit quality (see Fig. 2 ▸). These are the ‘reduced chisqr’, the ‘*Q* factor’ and the ‘*R* value’.

The ‘reduced chisqr’ value 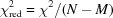 [see also equation (1)] provides a measure of fit quality across data sets and model configurations, with *N* the number of data points used for the fit and *M* the number of parameters of the fit model. 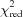 serves also as the optimization parameter in the least-squares optimization procedure. This value depends heavily on the quality of the data and how well the associated uncertainties were estimated. When representative uncertainty estimates are provided, a value of 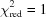 indicates that the data are described on average to within the uncertainties. However, if the uncertainties are excessively small (underestimated), or excessively large (overestimated), this condition no longer holds true. The value of the 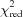 measure, therefore, is dependent on the quality of the uncertainty estimates.

The ‘*Q* factor’, defined as
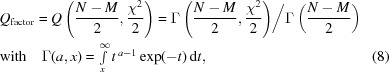
provides a second, independent measure of fit quality. It is the probability that a random set of *N* data points using the same model parameters would produce a 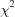 value equal to or higher than that obtained when using the real data set. For a fit of good quality, *Q*_factor_ should be in the range of 0.01–0.5 (the smaller the better), with a 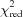 value of approximately 1.

In analogy to the *R* factor in crystallography (Hamilton, 1965 ▸, IUCr, 2008 ▸), *SASfit* also provides an ‘*R* value’ as quality criterion of a model in data analysis results:
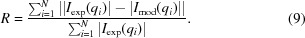
A value of *R* ranging between 0 and about 0.1 indicates a good to acceptable fit, whereas large values (up to infinity) denote a poor fit. It is especially important to realize that *R* is only a measure of precision and that it is not able to measure accuracy. Cases of data situations and model combinations that would be reported as false positives or negatives by the value of *R* are conceivable. Since the function being minimized is weighted by the uncertainties of the measured data [as can be seen in equation (10)], there is a weighted *R* value, ‘*wR* value’, provided (Hamilton, 1965 ▸), which takes those uncertainty estimates into account by
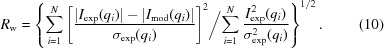



By providing several fit quality scores such as the *Q* factor and the *R* value next to the reduced chisqr value, *SASfit* assists the user in assessing the quality of each fit. Assessment of the quality of a fit using these measures is dependent on the provision of good uncertainty estimates. Recently, however, Franke *et al.* (2015 ▸) published a new goodness-of-fit test (‘correlation map’), which is intended to provide help for defining SAS fit quality when uncertainty estimates are not available.

### 3.5. Confidence in fitted parameter values   

In addition to the aforementioned fit quality estimators, *SASfit* provides confidence intervals for the fitted parameters and outputs the internal covariance matrix to enable the identification of highly dependent parameters, as shown in Fig. 4 ▸. (The respective menu ‘confidence intervals of fit parameters’ is accessible *via* the options menu of the model configuration window.) In order to find optimal model parameters, *SASfit* uses the Levenberg–Marquardt algorithm (Levenberg, 1944 ▸) to minimize the 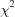 function [see equation (1)]. Internally, this algorithm approximates the Hessian matrix of the function subject to optimization.

The Hessian matrix provides a means to determine correlation coefficients of all pairs of fit parameters. The inverse of the Hessian matrix is the approximated formal covariance matrix *C* for the fit. The square root of diagonal elements 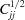 gives the standard deviation 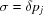 of the best-fit parameter 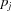, which holds only under the assumption that measurement errors are independent and normally distributed, and that the parameters are not correlated to each other. Recent work indicates that these assumptions are reasonably fulfilled (Franke *et al.*, 2015 ▸). Note that *SASfit* provides the standard deviation of the fit parameters, from which confidence intervals can be derived according, for example, to the *Guide to the Expression of Uncertainty in Measurements* (JCGM, 2008 ▸).

In order to assist the user in assessing the correlation of fit parameters, the correlation coefficient 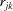 of every pair of fit parameters is shown by *SASfit* (see Fig. 4 ▸) in the upper triangular matrix in shades of red depending on their degree of correlation. For two parameters 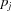 and 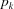 being optimized, the correlation coefficient 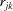 is given by
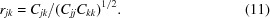



For uncorrelated parameters, 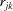 is expected to have a value close to zero, whereas for strongly correlated parameters 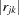 approaches one. Within the matrix, there is one row and one column associated with each parameter being optimized. They can be highlighted by clicking on a parameter entry in the lower half of the window. By selecting the row and column of two different fit parameters, their correlation coefficient 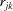 at the position of their common matrix element is highlighted.

When two parameters are strongly correlated it can happen that they both converge to unphysically large or small values during optimization. In this case, one has either to rewrite the form factor with fewer parameters or to fix one parameter to a value ideally determined using another technique. Another strategy would be to enhance the information content obtained from a SAS experiment, for example through contrast variation, potentially allowing for decoupling of the two strongly correlated parameters.

### 3.6. Data export   

Parameters and confidence values of the latest fit can be found under the ‘parameters of analytical size distrib.’ tab in the main window. It shows all of the configured model functions along with their parameters as ASCII text for easy export. The context menu (right click) has an option to write them to a semicolon-separated text file. This semicolon-separated text output consists of three columns for the size distribution, followed by three columns in the centre making up the form factor settings, with the final three columns reserved for the structure factor. In addition to the model parameters, the moments and other statistics calculated for the distribution function are given. Those values can be found under the tab ‘moments of analytical size distrib.’ and can be exported in the same manner as described above.

## 4. Batch processing   

**Figure 5:**
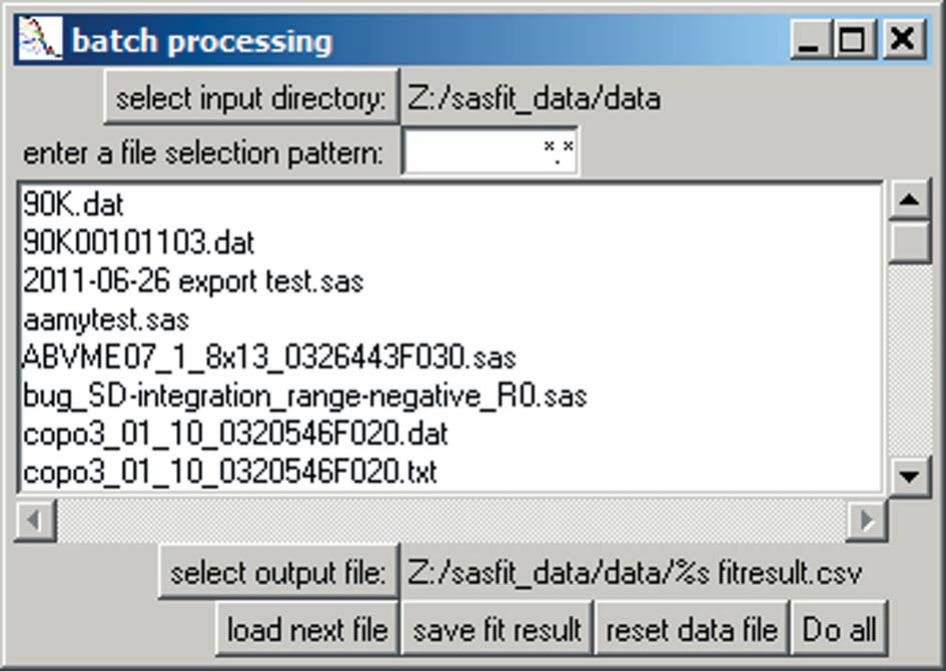
Panel for selection of data files for batch analysis and individual output file.

Once a model has been configured, it can be used for processing a batch of data files under ‘Options’ *→* ‘run batch’, as shown in Fig. 5 ▸. Hovering over the pattern input field reveals a short pop-up help text field on the pattern syntax for file selection. *SASfit* allows filtering of data file names from a user-defined input directory for model-dependent analysis.

## 5. Custom model functions as plug-ins   

In addition to the large library of existing model functions for form factors, structure factors and size distributions, *SASfit* features a flexible plug-in system which allows for custom model functions. It provides everything to enable users to write their own custom form factor and structure factor functions in the C programming language.

### 5.1. Plug-in concept   

In *SASfit*, a ‘plug-in’ is a container for model functions. It may contain an arbitrary number of form factors and structure factors. Both types are supported within a single plug-in at the same time, but it is recommended to use a plug-in for grouping model functions of a similar kind. In this way, a common set of internal helper routines not accessible publicly can be created and used for all model functions of a plug-in. *SASfit* plug-ins can be exchanged freely between different *SASfit* installations even in binary form, provided the PC platforms and architectures are compatible. To create new customized plug-ins, it is strongly recommended to build *SASfit* from its source code first. In this way, the build environment is verified to work correctly and the plug-in system compatibility is thus assured.

### 5.2. Retrieving the source code   

The latest source code of *SASfit*, including a history of all changes, can be obtained on the code-hosting page of the project (http://sasfit.org). There are two options to get the most recent source code: the recommended way is to use the distributed version control system *Mercurial* (https://mercurial.selenic.com) to ‘clone’ the project repository locally. This requires a third-party client for *Mercurial* to be installed but it simplifies the effort of updating to a new version. Alternatively, the complete source code of a given version or ‘snapshot’ can be downloaded. The technical details on the required build environment and the instructions for building the *SASfit* program on a specific platform can be found in the documentation (http://docs.sasfit.org).

### 5.3. Creating a new plug-in   

**Figure 6:**
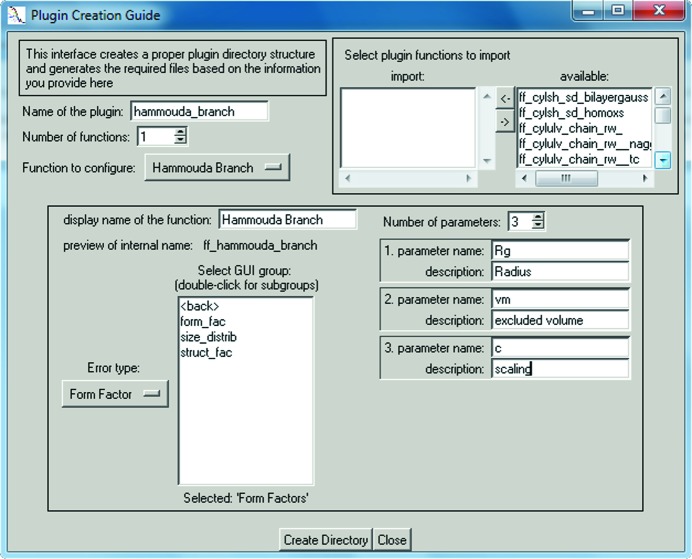
User interface for creating a new plug-in template, consisting of user-defined model functions, filled out according to the branched polymer example plug-in.

The first step in creating a new plug-in involves generating a new empty plug-in template containing a directory structure of source code skeleton files. For this, *SASfit* has to be run directly from the source code directory from which it was built and the plug-in guide shown in Fig. 6 ▸ has to be started. It can be found under the main menu ‘Tools’ → ‘create new plug-in’ and lets the user define the setup of a new plug-in function. The user is required to define a unique plug-in name, and at least one function has to be configured, including a descriptive name under which it can eventually be found in the model selection menu. Additionally, the plug-in guide expects the required parameters of each function to be defined. It is important to know the numerical implementation of the desired model function beforehand to determine the specific parameters needed. As existing model functions cannot be easily modified by the user, it is recommended to replicate a plug-in when modifications are required.

When created, new plug-in templates already contain the configured model functions, but lack any functionality and evaluate to zero. This ensures that the plug-in can be built right from the beginning by issuing the previously used build commands again. This will build only those source code files which are new or changed since the last run. In this case it will build the newly created empty plug-in only and add its binary files to the appropriate location automatically. To verify that the plug-in was built correctly *SASfit* has to be restarted, after which the new plug-in will be listed in the appropriate model selection list under ‘by plug-ins’.

### 5.4. Branched polymer plug-in function example   

Once the initial build of the new plug-in has been successful, it can be populated with the desired model implementation. The following example implements a single-polymer form factor for branched polymers formulated by Boualem Hammouda (2012 ▸).

By using a (mass) fractal model for the minimum path corresponding to the main chain backbone of the polymer, the form factor is described by
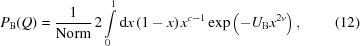
with the normalization factor being defined by
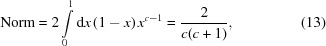
and the scattering variable 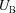 is expressed in terms of the radii of gyration 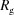:
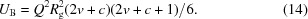
With a change of variable in 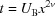 and 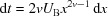 the integral 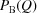 evaluates to
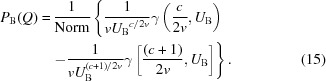
The remaining variables *v* for the excluded volume and *c* for the scaling factor become parameters of the model function next to the radii of gyration 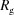. This formulation of the form factor can be translated into source code of the respective model function in a *SASfit* plug-in.

**Figure 7:**
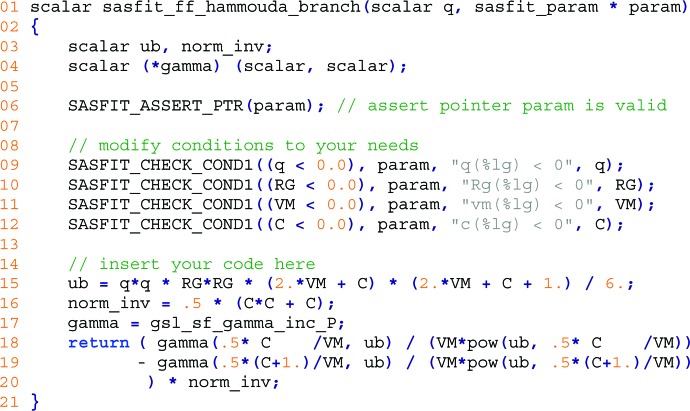
Source code of the branched copolymer form factor function of a custom *SASfit* plug-in.

The updated code shown in Fig. 7 ▸ replaces the automatically generated template source code of the function sasfit_ff_hammouda_branch() in the file sasfit_ff_hammouda_branch.c, which was generated by filling out the *SASfit* plug-in guide as shown in Fig. 6 ▸.

The function signature in line 1 was created by the plug-in guide along with the mandatory verification of input parameters in line 6. This function is evaluated for every individual *Q* value of the scattering vector provided in the first argument scalar q. Access to predefined input parameters of the model function is provided by automatically generated variables in upper case, RG, VM and C, along with range checks on them in lines 9–12 which were adjusted to a reasonable domain. Each range check consists of different parts. The first part is the condition which will raise an error; for example, in line 9 an error is to be raised if the scattering vector magnitude *q* is smaller than zero. The next part is the name of the common parameter, typically param. Subsequent parts of a check define an error message to be forwarded to the user. All variables that will be used are declared at the beginning of each model function. Line 3 in this example declares two floating point variables which will be defined later, while line 4 declares a short-cut name of a function which expects two input values. In line 17 this is set to a specific gamma function provided by the GNU Scientific Library (GSL; Galassi & Gough, 2009 ▸). The model function defined by the formula of Hammouda for branched polymers itself is implemented on lines 15–20, with the scattering variable 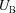 defined in line 15. The inversion of the normalization factor on line 16 replaces two divisions by multiplications in the final formula on line 20.

As demonstrated in the example, model functions in *SASfit* can make use of any function in the GSL but may also use a large set of predefined mathematical functions provided by *SASfit* directly. For example, a convenient wrapper sasfit_integrate() is available which simplifies usage of GSL integration routines by managing workspace memory in the background. Additionally, custom routines can make use of model functions defined in other plug-ins by declaring to import them during configuration with the plug-in guide. More information on plug-ins in *SASfit*, as well as an extensive guide on how to start writing custom models for *SASfit* on the Windows, Linux or MacOS platform complete with video guides, can be found online (http://docs.sasfit.org/Overview:_Plugins).

## 6. Example 2: characterization of a bimodal silica particle size distribution   

The interpretation of multimodal size distributions of nanoparticles is a demanding typical *SASfit* application. The procedure is demonstrated here by interpreting the scattering pattern of a bimodal size distribution of silica nanoparticles in aqueous solution. Recently, a suitable particle mixture was released as a certified reference material denoted ERM-FD-102 (Kestens & Roebben, 2014 ▸), which is commercially available as a European reference material. The intended use of ERM-FD-102 is the quality control and assessment of performance of nanoparticle size analysis methods, including SAXS. We have chosen ERM-FD-102 in order to allow all *SASfit* users to check our results easily and to verify the appropriate use of *SASfit*.

**Figure 8:**
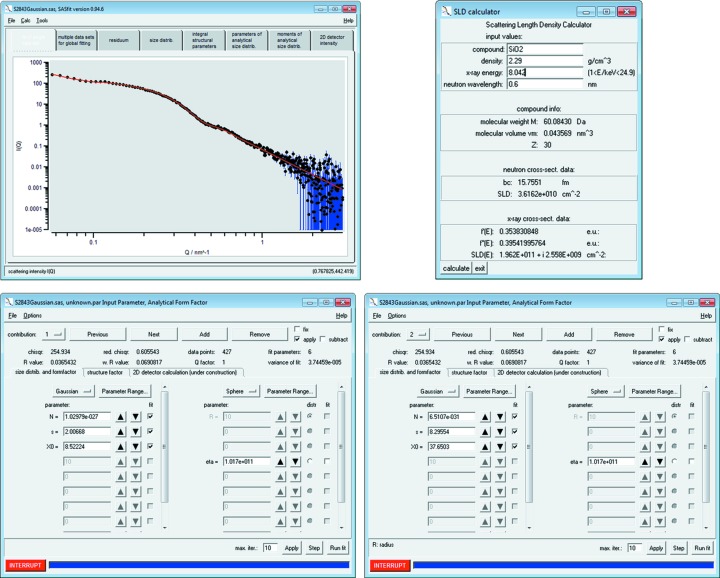
Upper left-hand figure: SAXS data of bimodal silica nanoparticles (European reference material ERM-FD-102) and a curve fitted using a model of spheres with Gaussian size distribution (black dots and red solid curve, respectively). The uncertainties of the intensity values are displayed as vertical blue lines. Upper right-hand figure: the scattering length density calculator provides a scattering length density of 1.962 × 10^11^ cm^−2^ for SiO_2_ particles with a density of 2.29 g cm^−3^. Lower figures: panels of the sphere form factor with Gaussian size distribution for the small particles (contribution 1, left-hand side) and large particles (contribution 2, right-hand side).

A sample volume of 20 µl was measured as received for 30 min on a commercial SAXS instrument. Its scattering intensity was converted to absolute scale using water as primary standard according to the procedure described by Orthaber *et al.* (2000 ▸) and was verified using a measurement of bovine serum albumin (Mylonas & Svergun, 2007 ▸). The resultant scattering curve with data in the range of *q*_min_ = 0.057 nm^−1^ to *q*_max_ = 3.0 nm^−1^ is shown in Fig. 8 ▸. [The obtained data set is provided as supplementary material (SilicaS2929.pdh) besides a preconfigured *SASfit* project file (SilicaS2929.sas).]

In the first step of data evaluation the scattering contrast ‘eta’ between the silica particles and the solvent is calculated. For this purpose we used the scattering length density calculator, which is available under the ‘Tools’ menu entry. The silica particle scattering length density is 1.962 × 10^11^ cm^−2^ by the stoichiometry of silica SiO_2_, the density of 2.29 (1) g cm^−3^ (Finsy *et al.*, 1985 ▸) and the copper 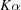 X-ray energy of 8.042 keV (see Fig. 8 ▸). In contrast to water, which has a scattering length density of 9.45 × 10^10^ cm^−2^, the sample scattering contrast is 1.017 × 10^11^ cm^−2^.

Next, a sphere model for the particles’ form factor with a Gaussian size distribution was chosen under the ‘Calc’ → ‘Single data set’ → ‘fit’ menu entry. Therein the value of the scattering contrast ‘eta’ was inserted as a fixed parameter for ‘contribution 1’ and for ‘contribution 2’. We then performed the fitting procedure described in the curve fitting workflow section (see also Fig. 3 ▸). The resultant best fit curve is shown together with the data points in Fig. 8 ▸ (red solid curve and points, respectively). The corresponding best fit values are displayed in the fit panels for the particle size contributions 1 and 2, respectively (lower row of Fig. 8 ▸). (The best fit values of the parameters and estimates of their uncertainties are displayed when clicking the button ‘parameters of analytical size distribution’.)

The parameters of the ERM-FD-102 sample are shown in Table 1 ▸. The estimate for the mean radius of silica particle class A is *X*_0_ = 8.52 (4) nm and that for class B is 37.65 (330) nm. These values are in good agreement with the number-weighted modal area-equivalent radii of 9.1 (8) and 42.0 (11) nm obtained by transmission and scanning electron microscopy (Kestens & Roebben, 2014 ▸). At first glance, it is surprising that the uncertainty of the mean radius is much larger for the larger particles (class B) than for the smaller ones. However, the particle size of class B is close to the instrumental limit of π/*q*_min_ = 56 nm, making its accurate determination more challenging, as also concluded in other work (Bressler *et al.*, 2015 ▸). In contrast, the radii of class A particles are far away from both the upper resolution limit and the low resolution limit of π/*q*_max_ = 1 nm. Accordingly, the uncertainty of the radii of class B becomes relatively large in comparison to that of the particles of class A.

The widths of the size distributions of class A and B are 2.00 (3) and 8.29 (304) nm, respectively, which are typical values for commercial silica particles. Also for *s*, the uncertainty for class B is larger than for class A for the same reason as for *X*_0_. It should be noted that number-weighted size distributions are important for the characterization of nanomaterials, which are defined by the European Commission as ‘a natural, incidental or manufactured material containing particles, in an unbound state or as an aggregate or as an agglomerate and where, for 50% or more of the particles in the *number size distribution*, one or more external dimensions is in the size range 1–100 nm’ (Potočnik, 2011 ▸).

Here, *SASfit* provides direct access to an estimate of number-weighted size distributions of nanoparticles. The implemented formula for curve fitting of spheres, 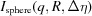, with Gaussian number-weighted size distribution, 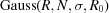, is
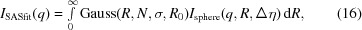
where the Gaussian size distribution is defined as
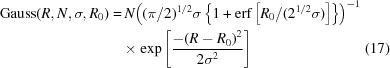
and the scattering of a sphere is given by
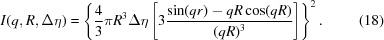



The approach to estimate the number-weighted distribution is only useful if the distribution is relatively narrow, typically smaller than 20% relative width. Alternatively, a very broad distribution has to be reasonably estimated by other means beforehand. In contrast, the recently published Monte Carlo approach for analysis of SAS data provides good estimates of volume-weighted size distribution but is much less suited for number-weighted determinations, owing to the lack of assumptions for the asymptotic behaviour of the distributions (Pauw *et al.*, 2013 ▸). *SASfit* calculates the number density distribution internally as long as the form factor is expressed in terms of a size, because it always contains the volume information. By default, the resulting distribution is plotted volume weighted instead for convenience. However, a different parametrization can be implemented if required; for example, a form factor of spheres can be defined to use input parameters of volume, after which volume-weighted Gaussian distribution parameters can be directly determined.

Following the procedure described in Example 1, the fitted *N* values of class A and B particles were converted to particle number concentrations of 1.02 (1) × 10^15^ and 6.51 (148) × 10^−11^ cm^−3^, respectively. The molar concentrations were 1.69 (2) × 10^-6^ and 1.08 (25) × 10^−9^ mol l^−1^. Therefore, the number ratio of small to large particles *N*_1_/*N*_2_ is 1567 (371). We also calculated the mass fractions assuming that the silica particles in our example have a density of 2.29 g cm^−3^ (Finsy *et al.*, 1985 ▸), resulting in 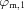 = 7.05 (7) mg g^−1^ for class A and 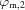 = 0.38 (9) mg g^−1^ for class B. Using the composition data given in the certification report of ERM-FD-102 (Kestens & Roebben, 2014 ▸) we calculated 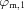 = 8.33 mg g^−1^ and 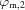 = 0.42 mg g^−1^. From these values, the mass ratio derived from *SASfit* 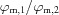 = 18.5 (46) is in good agreement with the values of 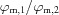 = 19.8 derived from the certification report. We conclude that the precision and accuracy of the *SASfit* parameters, and values derived thereof, are in good agreement with the reported values for the silica reference material.

## 7. Documentation   

A comprehensive manual is included with the software package (Kohlbrecher & Breßler, 2015 ▸). It contains the physical and mathematical details and definitions of the internal algorithms, as well as documentation, references and implementation notes for most of the models. Additionally, there is a collaborative wiki web site available, containing further information on installation details and providing help for setting up and writing custom plug-ins. For core topics in using the *SASfit* program there are also video guides available online (https://www.youtube.com/user/SASfitTeam). The numerical part of *SASfit* is written in C and the user interface in Tcl/Tk. The latest packages are available at the project page (http://sasfit.org/files/0.94.6).

## 8. Future outlook   

In addition to the developments for general use elaborated upon here, an extensive effort is underway to implement a new solver for the Ornstein–Zernike equation for different closure relations and potentials. A specialized user interface plots the numerical solution which can be used for structure factor input in model dependent analysis. The details of the solver and its implementation as well as its usage will be presented in a future publication.

Since the early versions of the *SASfit* program, the Levenberg–Marquardt (Levenberg, 1944 ▸) algorithm has been used to find solutions for multi-dimensional nonlinear optimization problems. Users often experience stability issues or sometimes even crashes of the optimization routine of *SASfit*, in particular when optimizing several parameters of a complex model at once. Those issues may be caused either by correlated parameters within a model (though these are hard to predict) or by instabilities of the optimization algorithm. Further development of the *SASfit* software will, therefore, provide alternative optimization algorithms. These will include modern versions of the Levenberg–Marquardt algorithm with improved numerical stability over the original implementation. Additional options for parameter constraints will improve the overall fit stability, in contrast to the current implementation which interrupts a fit if parameter values leave their defined range. Better minimization routines may be able to automatically account for that as well. This will improve the overall workflow and user experience with the *SASfit* analysis program.

## Supplementary Material

Data set and project file for RM-8011. DOI: 10.1107/S1600576715016544/vg5026sup1.zip

Data set and project file for ERM-FD-102. DOI: 10.1107/S1600576715016544/vg5026sup2.zip

## Figures and Tables

**Table 1:** Parameters of silica nanoparticles ERM-FD-102 fitted with a bimodal Gaussian size distribution The population of small particles is labelled as ‘Particle class A’ and that of the large particles as ‘Particle class B’ in accordance with the ERM-FD-102 certification report (Kestens Roebben, 2014 ▸). Fit parameters are the particle number *N*, the mean radius *X*_0_ and the width of the size distribution *s*. The values of the mass fraction of the particles 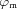 are given as derived from the *SASfit* parameter *N* and calculated from the data given in the certification report.

Parameter	Particle class A Contribution 1	Particle class B Contribution 2
*N* (cm^3^, moll^1^ )	1.02(1) 10^15^	6.51 (148) 10^11^
	1.69 (2) 10^6^	1.08 (25) 10^9^
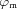 (*SASfit*) (mg g^1^)	7.05 (7)	0.38 (9)
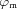 (certification report)† (mg g^1^)	8.33	0.42
Mean radius *X*_0_ (*SASfit*) (nm)	8.52 (4)	37.65 (330)
Mean radius (certification report) (nm)‡	9.1 (8)	42.0 (11)
Width of distribution *s* (nm)	2.00 (3)	8.29 (304)

Number ratio *N*_1_/*N*_2_	1567 371 ( 24%)	
Mass ratio 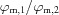 (*SASfit*)	18.5 4.6 ( 25%)	
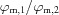 (certification report)	19.8	

† Values were calculated from the information on the production data given in the certification report of ERM-FD-102 (Kestens Roebben, 2014 ▸).

‡ Number-weighted modal area-equivalent diameter as obtained by transmission and scanning electron microscopy (Kestens Roebben, 2014 ▸).

## References

[bb1] Bevington, P. R. & Robinson, D. K. (2003). *Data Reduction and Error Analysis for the Physical Sciences*, 3rd ed. New York: McGraw-Hill Higher Education.

[bb2] Brandt, B. van den, Glättli, H., Hautle, P., Kohlbrecher, J., Konter, J. A., Michels, A., Stuhrmann, H. B. & Zimmer, O. (2007). *J. Appl. Cryst.* **40**, s106–s110.

[bb3] Bressler, I., Pauw, B. R. & Thünemann, A. F. (2015). *J. Appl. Cryst.* **48**, 962–969. 10.1107/S1600576715007347 PMC4453982 26089769

[bb4] De Temmerman, P. J., Verleysen, E., Lammertyn, J. & Mast, J. (2014). *J. Nanopart. Res.* **16**, 2628.

[bb5] Finsy, R., Moreels, E., Bottger, A. & Lekkerkerker, H. (1985). *J. Chem. Phys.* **82**, 3812–3816.

[bb6] Förster, S., Apostol, L. & Bras, W. (2010). *J. Appl. Cryst.* **43**, 639–646.

[bb7] Franke, D., Jeffries, C. M. & Svergun, D. I. (2015). *Nat. Methods*, **12**, 419–422. 10.1038/nmeth.3358 25849637

[bb8] Galassi, M. & Gough, B. (2009). *Gnu Scientific Library: Reference Manual*, 3rd ed. Network Theory.

[bb9] Gleber, G., Cibik, L., Haas, S., Hoell, A., Müller, P. & Krumrey, M. (2010). *J. Phys. Conf. Ser.* **247**, 012027.

[bb10] Hamilton, W. C. (1965). *Acta Cryst.* **18**, 502–510.

[bb11] Hammouda, B. (2012). *Macromol. Theory Simul.* **21**, 372–381.

[bb12] Ilavsky, J. & Jemian, P. R. (2009). *J. Appl. Cryst.* **42**, 347–353.

[bb13] IUCr (2008). *R Factor*, http://reference.iucr.org/dictionary/R_factor.

[bb20] JCGM (2008). *Evaluation of Measurement Data – Guide to the Expression of Uncertainty in Measurement*, JGM100:2008. Joint Committee for Guides in Metrology.

[bb14] Kaiser, D. L. & Watters, R. L. (2007). Reference Material RM-8011. National Institute of Standards and Technology, Gaithersburg, MD, USA.

[bb15] Kestens, V. & Roebben, G. (2014). Reference Material ERM-FD-102. European Commission, Joint Research Centre, Institute for Reference Materials and Measurements (IRMM), Geel, Belgium.

[bb16] Kohlbrecher, J. & Breßler, I. (2015). *SASfit Manual*, http://kur.web.psi.ch/sans1/SANSSoft/sasfit.pdf.

[bb17] Kohlbrecher, J., Buitenhuis, J., Meier, G. & Lettinga, M. P. (2006). *J. Chem. Phys.* **125**, 044715. 10.1063/1.2220564 16942182

[bb18] Levenberg, K. (1944). *Q. Appl. Math.* **2**, 164–168.

[bb19] Meli, F., Klein, T., Buhr, E., Frase, C. G., Gleber, G., Krumrey, M., Duta, A., Duta, S., Korpelainen, V., Bellotti, R., Picotto, G. B., Boyd, R. D. & Cuenat, A. (2012). *Meas. Sci. Technol.* **23**, 125005.

[bb21] Mylonas, E. & Svergun, D. I. (2007). *J. Appl. Cryst.* **40**, s245–s249.

[bb22] Orthaber, D., Bergmann, A. & Glatter, O. (2000). *J. Appl. Cryst.* **33**, 218–225.

[bb23] Pauw, B. R., Pedersen, J. S., Tardif, S., Takata, M. & Iversen, B. B. (2013). *J. Appl. Cryst.* **46**, 365–371. 10.1107/S0021889813001295 PMC3627408 23596341

[bb24] Petoukhov, M. V., Franke, D., Shkumatov, A. V., Tria, G., Kikhney, A. G., Gajda, M., Gorba, C., Mertens, H. D. T., Konarev, P. V. & Svergun, D. I. (2012). *J. Appl. Cryst.* **45**, 342–350. 10.1107/S0021889812007662 PMC4233345 25484842

[bb25] Potočnik, J. (2011). *Commission Recommendation of 18 October 2011 on the Definition of Nanomaterial*, pp. 38–40. Brussels: European Commision.

[bb200] Sztucki, M., Narayanana, T. & Beaucage, G. (2007). *J. Appl. Phys.* **101**, 114304.

[bb26] Varga, Z., Yuana, Y., Grootemaat, A. E., van der Pol, E., Gollwitzer, C., Krumrey, M. & Nieuwland, R. (2014). *J. Extracell. Vesicles*, **3**, 23298. 10.3402/jev.v3.23298 PMC3916677 24511372

[bb27] Vavrin, R., Kohlbrecher, J., Wilk, A., Ratajczyk, M., Lettinga, M. P., Buitenhuis, J. & Meier, G. (2009). *J. Chem. Phys.* **130**, 154903. 10.1063/1.3103245 19388768

[bb28] Wiedenmann, A., Gahler, R., Dewhurst, C. D., Keiderling, U., Prevost, S. & Kohlbrecher, J. (2011). *Phys. Rev. B*, **84**, 214303.

